# Sex‐Based Body Composition Changes in Patients With Rectal Cancer Undergoing Preoperative Chemoradiotherapy: A Prospective Observational Study

**DOI:** 10.1002/ags3.70231

**Published:** 2026-05-05

**Authors:** Shinya Abe, Hiroaki Nozawa, Kentaro Abe, Yumi Yokota, Shunsuke Hori, Yusuke Taguchi, Mitsutaka Yakabe, Kazuhito Sasaki, Sumito Ogawa, Soichiro Ishihara

**Affiliations:** ^1^ Department of Surgical Oncology, Graduate School of Medicine The University of Tokyo Bunkyo‐ku Tokyo Japan; ^2^ Department of Surgery The Fraternity Memorial Hospital Sumida‐ku Tokyo Japan; ^3^ Department of Geriatric Medicine, Graduate School of Medicine The University of Tokyo Bunkyo‐ku Tokyo Japan

**Keywords:** body composition, chemoradiotherapy, rectal cancer, sex difference

## Abstract

**Introduction:**

Preoperative chemoradiotherapy (CRT) for rectal cancer may have an attritional impact on body composition and functional status, affecting postoperative outcomes. This prospective study characterized changes in body composition, muscle strength, and physical performance in patients with rectal cancer receiving CRT.

**Methods:**

Body composition measures by a bioelectrical impedance analysis, hand grip strength (HGS), gait speed, and the nutritional status by the Mini‐Nutritional Assessment (MNA) were assessed at Weeks 0 (pre‐CRT), 2, 4, 6, and 10 (post‐CRT). The pre‐sarcopenic status was defined as low muscle mass alone and the sarcopenic status as low muscle mass and low muscle strength or function.

**Results:**

The median age of 38 enrolled patients (25 men) was 59 years. The mean body fat mass, phase angle, and non‐dominant HGS significantly decreased by 1.3 kg, 0.3°, and 0.7 kg, respectively, after CRT. Skeletal muscle mass and the MNA slightly decreased, while gait speed did not change. Significant reductions were observed in body fat mass (2.3 kg, *p* = 0.04) and non‐dominant HGS (1.3 kg, *p* < 0.01) in women, while slight decreases were noted in men. Although no significant changes were detected in the pre‐ and post‐CRT sarcopenic status in men (4 pre‐sarcopenic patients), the numbers of pre‐sarcopenic and sarcopenic female patients increased from pre‐CRT (1 and 0) to post‐CRT (1 and 2).

**Conclusion:**

Muscle strength and body fat decreased in patients receiving CRT, particularly women. These exploratory data provide a foundation for future investigations into the clinical utility of sex‐sensitive therapeutic approaches.

## Graphical abstract

This prospective study observed that preoperative chemoradiotherapy for rectal cancer was associated with reductions in body fat and grip strength, which appeared to be more pronounced in female patients. These findings suggest that sex‐sensitive nutritional and exercise support may be beneficial to help maintain muscle mass and physical function during treatment.

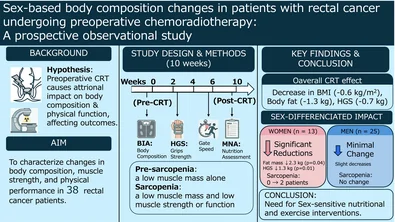

## 1 Introduction

Clinical trials have shown that long‐course chemoradiotherapy (CRT) plus chemotherapy, namely, total neoadjuvant therapy, contributed to an improved prognosis in patients with locally advanced rectal cancer [1, 2], and long‐course CRT is an important component of this treatment strategy.

Previous studies demonstrated that body composition at post‐CRT and body composition changes during preoperative CRT are related to short‐ and long‐term outcomes [3, 4, 5]. A decrease in subcutaneous fat during preoperative CRT and post‐CRT has been associated with downstaging and the tumor regression grade [6]. Additionally, skeletal muscle mass loss during preoperative CRT [3] and a low skeletal muscle mass at post‐CRT have been identified as poor prognostic factors [4]. Therefore, investigating the impact of changes in body composition during CRT on short‐ and long‐term outcomes and identifying areas for improvement are expected to achieve better therapeutic outcomes for locally advanced rectal cancer in the future. However, these body composition changes have predominantly been assessed using computed tomography (CT), and the effects of changes in other body composition measures, including the phase angle, intracellular water, and extracellular water (ECW), obtained using a bioelectrical impedance analysis (BIA), have yet to be examined.

Although skeletal muscle mass loss has been shown to have a negative impact on the oncologic outcomes of several cancers, including rectal cancer [5, 7], it is an essential criterion in the sarcopenia criteria proposed by the European Working Group on Sarcopenia in Older People and the Asian Working Group on Sarcopenia [8, 9]. Other sarcopenia criteria include muscle strength and physical function. Previous studies investigated changes in body composition, muscle strength, and physical function during neoadjuvant therapy for a number of cancer types [10]; however, limited information is currently available for locally advanced rectal cancer.

Sex differences have been shown to affect the occurrence of adverse events in patients receiving anticancer drug treatments [11]. A pooled analysis of 4 randomized controlled trials on Japanese patients with stage III colorectal cancer reported that female sex was associated with a higher incidence of Grade 3–4 adverse events [12]. Our findings also indicated that adjuvant chemotherapy for patients with colorectal cancer had a negative impact on postoperative recovery in women [13]. Although the mechanisms underlying the relationships between sex differences and adverse events induced by anticancer drugs as well as body composition changes remain unclear, we hypothesized that sex differences may affect body composition due to higher rates of adverse events during CRT in female patients.

Therefore, we herein prospectively investigated body composition during preoperative CRT for locally advanced rectal cancer and also examined changes in body composition during the treatment with a focus on sex differences.

## 2 Methods

### 2.1 Study Design

This prospective observational study was approved by the Ethics Committee of the University of Tokyo (No: 2021363NI). Written informed consent was obtained from all individual participants included in the study. We started to recruit rectal cancer patients who received preoperative CRT in March 2022. All patients with locally advanced tumors (cT3‐4/any N/M0 or any T/N+/M0) below the peritoneal reflection or a rectal tumor reaching the peritoneal reflection in a pretreatment examination were analyzed. To evaluate the actual in‐practice status, the diagnostic metrics of sarcopenia were assessed in all patients regardless of whether they had completed the scheduled treatment. Patients were followed up at our hospital until Jan 31, 2025. Measurements were performed at Weeks 0 (pre‐CRT), 2, 4, 6, and 10 (post‐CRT).

### 2.2 CRT Regimen

All patients received preoperative radiotherapy with radiation (55 Gy in 25 fractions for 5 weeks) and were concurrently administered 5‐fluorouracil (5‐FU) and irinotecan [14]. Tegafur/uracil (300 mg/m^2^/day) and calcium folinate (75 mg/body weight/day) were administered orally 3 times daily on Days 1–5, 8–12, 15–19, 22–26, and 29–33. Irinotecan (80 mg/m^2^) was administered as an intravenous infusion on Days 1, 15, 29, and 43. Adverse events were graded according to the Common Terminology Criteria for Adverse Events from the National Cancer Institute (version 5.0).

### 2.3 Nutritional Assessment

The risk of malnutrition was screened using the Mini‐Nutritional Assessment (MNA), which comprises 18 questionnaires under four different domains with a maximum score of 30. Scores < 17 indicate a risk of undernutrition [15].

### 2.4 Gait Speed

Gait speed may be used as one of the parameters to assess physical performance. In the present study, gait speed was calculated as the time to walk 6 m during a 10‐m trial. Gait speed was shown as the mean of the two trials.

### 2.5 Hand Grip Strength

Hand grip strength (HGS), one of the assessments for muscle strength, was measured using a digital hand dynamometer (EKJ077; EVERNEW Inc., Tokyo, Japan). The test was repeated twice for each hand, alternating sides. Mean HGS for the dominant or non‐dominant side was calculated in kg.

### 2.6 Body Composition Analysis Using a BIA

Body composition was measured in the standing position using the multifrequency BIA, InBody 770 (InBody Japan Inc., Tokyo, Japan).

Patients held retractable electrodes while standing barefoot on the other electrodes on the device platform. Estimates of body composition, such as body fat mass, total skeletal muscle mass, phase angle, ECW, and total body water (TBW), were calculated using the data management software, Lookin'Body120 (InBody Japan Inc., Tokyo, Japan), connected to the multifrequency BIA.

### 2.7 Sarcopenia

Sarcopenia was defined according to consensus criteria [9], namely, a low muscle mass (skeletal muscle index [SMI] < 7.0 kg/m^2^ in men and < 5.7 kg/m^2^ in women) with either low muscle strength (HGS < 28 kg for men or < 18 kg for women) or low physical performance (6‐m walk speed < 1.0 m/s). The HGS value used to classify sarcopenia was calculated as the mean of the dominant and non‐dominant HGS values. The pre‐sarcopenic status was defined as a low muscle mass without low muscle strength or low physical performance; the sarcopenic status as a low muscle mass with low muscle strength or low physical performance; and severe sarcopenia as a low muscle mass, low muscle strength, and low physical performance.

### 2.8 Collection of Other Data

The following clinicopathological variables were retrieved from our prospectively collected database: sex, age, the body mass index (BMI), Charlson comorbidity index, primary tumor location, and histology prior to CRT. Clinical staging was performed according to the TNM classification system, as outlined in the Japanese Society for Cancer of the Colon and Rectum guidelines [16].

### 2.9 Statistical Analysis

All continuous variables are presented as medians and interquartile ranges (IQR) or as the mean and standard error of the mean (SE). Categorical variables were analyzed using the chi‐square test with Yates correction when appropriate or Fisher's exact test, and continuous variables were analyzed using the Wilcoxon rank‐sum test or Kruskal–Wallis test. Patients with incomplete body composition records due to treatment‐related adverse events, such as fatigue and anorexia (*n* = 8 for males; *n* = 3 for females), were excluded from the longitudinal analysis. Consequently, the results presented in Figure 2 were constructed using a complete‐case analysis approach to ensure data consistency across the treatment period. All analyses were performed using JMP Pro 17.2 software (SAS Institute Inc., Cary, NC, USA). *p* values < 0.05 were considered to be significant.

## 3 Results

### 3.1 Clinical Characteristics

The median age of 38 patients (25 men) was 59 years (IQR, 52–69 years). Table 1 shows baseline characteristics for the entire cohort; eight patients (21.1%) had a Charlson comorbidity index ≥ 1. The clinical T‐stage was mostly T3 (81.6%) and T4 (15.8%), and 15 patients (39.5%) had swollen lateral lymph nodes. A comparison between men and women showed similar patient‐related factors, including BMI and the Charlson comorbidity index. The incidence of each tumor‐related factor was similar between the two sexes. The mean relative dose intensity of irinotecan was 91.0% in men and 83.5% in women, with no significant difference (*p* = 0.31).

**TABLE 1:** Baseline characteristics.

Variables	All (*n* = 38)	Men (*n* = 25)	Women (*n* = 13)	*p*
Patient factors				
Age (years)^a^	59 (52–69)	58 (50–63)	64 (54–73)	0.15
BMI (kg/m^2^)^a^	22.7 (20.3–24.2)	22.1 (20.3–24.4)	22.7 (19.4–26.7)	0.76
Charlson comorbidity index				
0	30 (78.9)	19 (76.0)	11 (84.6)	0.78
1	3 (7.9)	2 (8.0)	1 (7.7)	
2	4 (10.6)	3 (12.0)	1 (7.7)	
4	1 (2.6)	1 (4.0)	0	
Tumor factors				
Distance from AV (cm)^a^	5.0 (3.0–6.0)	4.0 (1.5–7.0)	5.0 (4.0–6.0)	0.20
Clinical T‐stage				
cT2	1 (2.6)	0	1 (7.7)	0.33
cT3	31 (81.6)	21 (84.0)	10 (76.9)	
cT4	6 (15.8)	4 (16.0)	2 (15.4)	
Clinical MesoLN				
Negative	8 (21.1)	5 (20.0)	3 (23.1)	0.83
Positive	30 (78.9)	20 (80.0)	10 (76.9)	
Clinical LLN				
Negative	23 (60.5)	17 (68.0)	6 (46.2)	0.19
Positive	15 (39.5)	8 (32.0)	7 (53.8)	
Histopathological type				
Differentiated (well/moderate)	35 (92.1)	34 (94.4)	13 (100)	0.10
Others	3 (7.9)	2 (5.6)	0	
Relative dose intensity of chemoradiotherapy				
Irinotecan^b^		91.0 (20.3)	83.5 (22.4)	0.31
Tegafur‐uracil/calcium folinate^b^		97.8 (1.5)	97.3 (2.0)	0.83
Radiation^b^		99.6 (2.1)	98.4 (5.9)	0.36

*Note:* Values in parentheses are percentages, unless indicated otherwise.

Abbreviations: AV, anal verge; LLN, lateral lymph node; MesoLN, mesorectal lymph node.

^a^ Values are medians (interquartile ranges).

^b^ Values are means (standard deviations).

Of the 25 male patients, 25, 25, 17, 21, and 25 underwent body composition measurements at weeks 0 (pre‐CRT), 2, 4, 8, and 10 (post‐CRT), respectively. Of the 13 female patients, 13, 12, 12, 10, and 13 underwent these measurements at weeks 0, 2, 4, 8, and 10, respectively. The reasons for missing measurements were mostly physical adverse events such as fatigue and anorexia. Table 2 shows a comparison of adverse event grades between the sexes. Neutropenia was significantly more prevalent in women (*p* = 0.020). Additionally, AST and ALT abnormalities were generally more prevalent in women than in men. Severe physical adverse events were also more frequent in women than in men.

**TABLE 2:** Relationships between sex differences and adverse events.

Adverse events	Men (*n* = 25)	Women (*n* = 13)	*p*
All			
Grade 0	0	0	0.19
Grade 1–2	17 (68)	6 (46.2)	
Grade 3	8 (32)	7 (53.8)	
Leukopenia			
Grade 0	21 (84)	10 (76.9)	0.33
Grade 1–2	4 (16)	2 (15.4)	
Grade 3	0	1 (7.7)	
Neutropenia			
Grade 0	23 (92)	7 (53.8)	0.020
Grade 1–2	2 (8)	5 (38.5)	
Grade 3	0	1 (7.7)	
Anemia			
Grade 0	16 (64)	5 (38.5)	0.15
Grade 1–2	9 (36)	7 (53.8)	
Grade 3	0	1 (7.7)	
AST abnormality			
Grade 0	22 (88)	8 (61.5)	0.059
Grade 1–2	2 (8)	5 (38.5)	
Grade 3	1 (4)	0	
ALT abnormality			
Grade 0	20 (80)	7 (53.8)	0.062
Grade 1–2	5 (20)	4 (30.8)	
Grade 3	0	2 (15.4)	
Diarrhea			
Grade 0	4 (8)	0	0.16
Grade 1–2	14 (56)	9 (69.2)	
Grade 3	7 (28)	4 (30.8)	
Nausea			
Grade 0	17 (68)	7 (53.8)	0.68
Grade 1–2	7 (28)	5 (38.5)	
Grade 3	1 (4)	1 (7.7)	
Anorexia			
Grade 0	20 (80)	8 (61.5)	0.39
Grade 1–2	3 (12)	2 (15.4)	
Grade 3	2 (8)	3 (23.1)	
Fatigue			
Grade 0	24 (96)	10 (76.9)	0.088
Grade 1–2	1 (4)	1 (7.7)	
Grade 3	0	2 (15.4)	
Anorectal pain			
Grade 0	5 (20)	5 (38.5)	0.13
Grade 1–2	20 (80)	7 (53.8)	
Grade 3	0	1 (7.7)	
Body weight loss			
Grade 0	16 (64)	6 (46.2)	0.29
Grade 1–2	9 (36)	7 (53.8)	
Grade 3	0	0	

*Note:* Values in parentheses are percentages, unless indicated otherwise.

### 3.2 Measures of Anthropometry

Table 3 shows body composition changes during CRT. Among the study cohort, BMI (23.0 ± 0.69 kg/m^2^ vs. 22.4 ± 0.63 kg/m^2^, *p* = 0.024), body fat mass (14.7 ± 1.4 kg vs. 13.4 ± 1.3 kg, *p* = 0.018), and phase angle (4.9° ± 0.1° vs. 4.6° ± 0.2°, *p* < 0.0001) significantly decreased after CRT. While BMI (24.0 ± 1.7 kg/m^2^ vs. 22.9 ± 1.5 kg/m^2^, *p* = 0.054) and body fat mass (18.5 ± 3.2 kg vs. 16.2 ± 2.9 kg, *p* = 0.038) decreased in women, reductions in BMI and body fat mass were not significant in men. SMI, skeletal muscle mass, and the ECW/TBW ratio remained unchanged in both sexes.

**TABLE 3:** Changes in body composition from pre‐ to post‐neoadjuvant chemoradiotherapy.

Variables	All (*n* = 38)		*p*	Men (*n* = 25)		*p*	Women (*n* = 13)		*p*
	Pre‐CRT	Post‐CRT		Pre‐CRT	Post‐CRT		Pre‐CRT	Post‐CRT	
BMI (kg/m^2^)	23.0 (±0.7)	22.4 (±0.6)	0.024	22.5 (±0.6)	22.2 (±0.6)	0.23	24.0 (±1.7)	22.9 (±1.5)	0.054
Body fat mass (kg)	14.7 (±1.4)	13.4 (±1.3)	0.018	12.7 (±1.1)	11.9 (±1.2)	0.28	18.5 (±3.2)	16.2 (±2.9)	0.038
SMI (kg/m^2^)	7.2 (±0.2)	7.2 (±0.2)	0.75	7.7 (±0.2)	7.8 (±0.1)	0.21	6.3 (±0.1)	6.1 (±0.2)	0.48
Skeletal muscle mass (kg)	26.5 (±1.0)	26.2 (±1.0)	0.16	29.7 (±0.9)	29.4 (±0.9)	0.36	20.3 (±0.1)	19.9 (±0.1)	0.18
Phase angle (°)	4.9 (±0.1)	4.6 (±0.2)	< 0.0001	5.1 (±0.2)	4.9 (±0.19)	0.002	4.4 (±0.1)	3.9 (±0.1)	0.001
ECW/TBW ratio	0.4 (±0.01)	0.4 (±0.01)	0.13	0.4 (±0.002)	0.4 (±0.002)	0.28	0.4 (±0.005)	0.4 (±0.01)	0.11

*Note:* Values are the mean ± standard error of the mean.

Abbreviations: BMI, body mass index; ECW, extracellular water; N/A, not applicable; SMI, skeletal muscle index; TBW, total body water.

**FIGURE 1:**
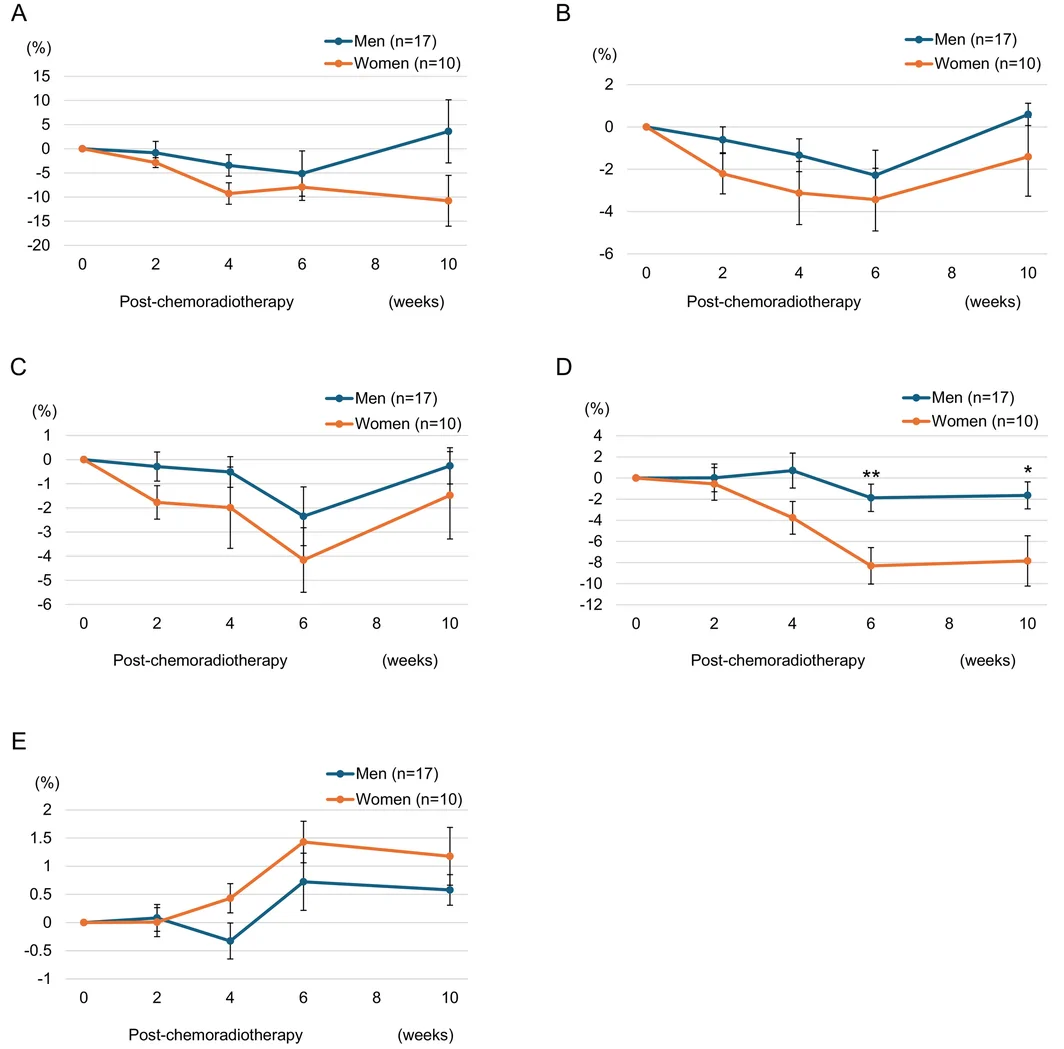
Magnitudes of changes in body composition parameters in patients with lower rectal cancer assessed by a bioelectrical assessment during preoperative CRT. (A) Body fat mass, (B) Skeletal muscle index, (C) Skeletal muscle mass, (D) Phase angle, and (E) Extracellular water/total body water ratio. *p < 0.05, **p < 0.05 significant difference between the sexes.

Figure 1 shows relative changes in body composites, normalized to baseline values, in patients who participated in all examination sessions (17 men and 10 women). Regarding longitudinal measurements, missing data rates were 32.0% (8/25) for males and 23.1% (3/13) for females. A comparison of baseline characteristics between patients with and without missing measurements showed no significant differences (Table S1). Body fat mass, SMI, skeletal muscle mass, and the phase angle decreased in men between 0 and 6 weeks, and then recovered to baseline values by Week 10. Similar results were obtained for SMI and skeletal muscle mass in women; however, body fat mass and the phase angle did not recover at Week 10. The magnitude of the percent change in the phase angle at Weeks 6 and 10 was significantly larger in women than in men (*p* = 0.009 at Week 6 and *p* = 0.04 at Week 10). The change in the ECW/TBW ratio was similar in both sexes.

### 3.3 Measures of Nutrition, Muscle Strength and Physical Function

**FIGURE 2:**
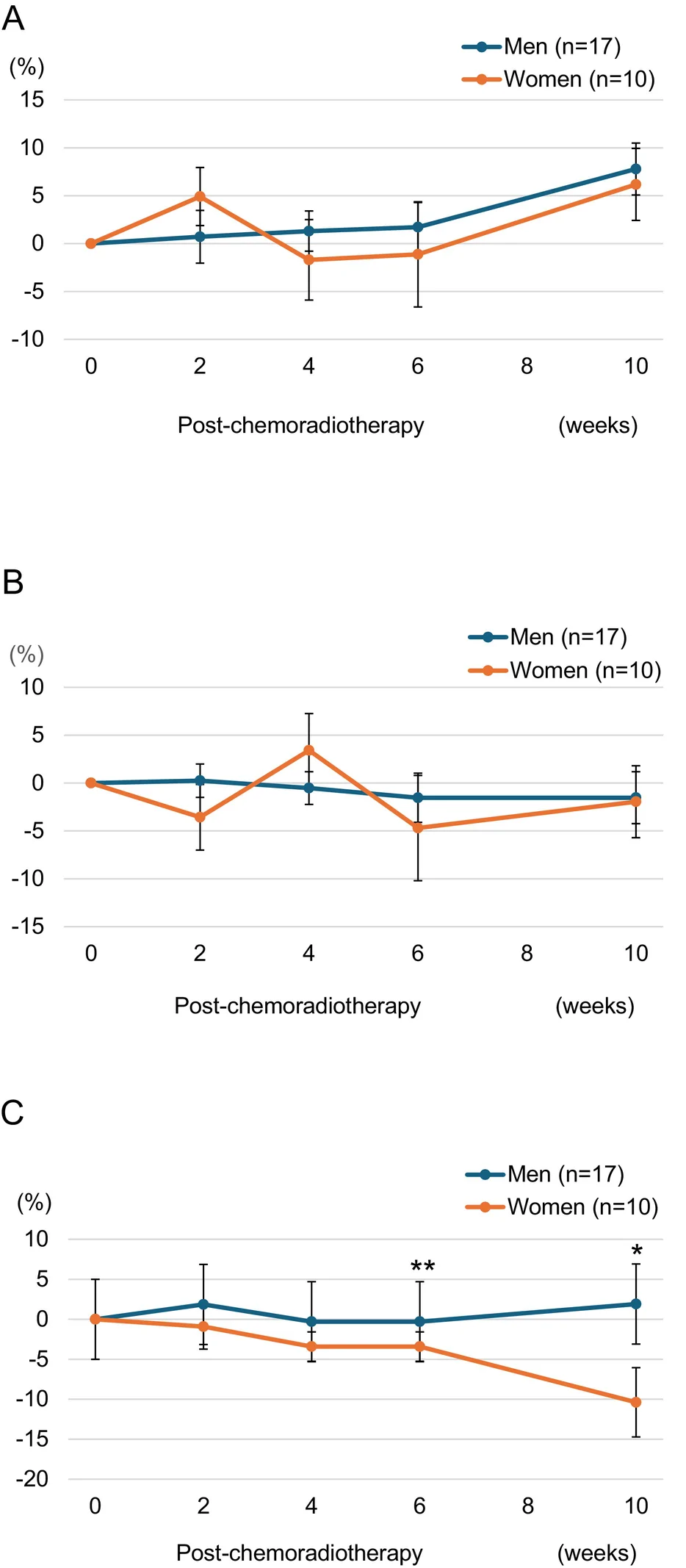
Magnitudes of changes in physical and muscle function parameters in patients with lower rectal cancer assessed during preoperative CRT. (A) Gait speed, (B) dominant hand grip strength, and (C) non‐dominant hand grip strength. *p < 0.05, **p < 0.01 significant difference between the sexes.

The MNA scores at pre‐CRT and post‐CRT are shown in Table 4. CRT caused a little reduction in the score in both men and women. Table 4 also shows changes in gait speed and dominant and non‐dominant HGS post‐CRT. Although mean gait speed and dominant HGS did not significantly change, mean non‐dominant HGS significantly decreased post‐CRT (29.3 ± 1.3 kg vs. 28.6 ± 1.5 kg, *p* = 0.018). No significant change was noted in dominant or non‐dominant HGS in men. However, non‐dominant HGS significantly decreased in women (20.6 ± 0.9 kg vs. 18.8 ± 1.1 kg, *p* = 0.0005). Figure 2 shows relative changes in gait speed and HGS, normalized to baseline values, in patients who participated in all examination sessions. The magnitudes of the percent changes in gait speed and dominant HGS were similar in both sexes. However, non‐dominant HGS decreased at Weeks 6 and 10 in women, and differences in the magnitude of the ratio in non‐dominant HGS between sexes at Weeks 6 (*p* = 0.005) and 10 (*p* = 0.014) were significant.

**TABLE 4:** Changes in nutrition and muscle functions from pre‐ to post‐neoadjuvant chemoradiotherapy.

Variables	All (*n* = 38)		*p*	Men (*n* = 25)		*p*	Women (*n* = 13)		*p*
	Pre‐CRT	Post‐CRT		Pre‐CRT	Post‐CRT		Pre‐CRT	Post‐CRT	
MNA	23.4 (±0.7)	22.7 (±0.7)	0.40	24.3 (±1.2)	23.5 (±1.2)	0.38	21.9 (±0.94)	21.4 (±1.1)	0.93
Gait speed (m/s)	1.2 (±0.03)	1.3 (±0.03)	0.061	1.2 (±0.03)	1.3 (±0.04)	0.068	1.3 (±0.06)	1.2 (±0.05)	0.68
Dominant HGS (kg)	31.2 (±1.4)	30.3 (±1.5)	0.35	36.0 (±1.2)	35.4 (±1.4)	0.35	21.8 (±0.8)	20.5 (±0.9)	0.22
Non‐dominant HGS (kg)	29.3 (±1.3)	28.6 (±1.5)	0.018	33.9 (±1.2)	33.7 (±1.2)	0.42	20.6 (±0.9)	18.8 (±1.1)	0.0005

*Note:* Values are the mean ± standard error of the mean.

Abbreviation: HGS, hand grip strength.

Using consensus diagnostic sarcopenia criteria [9], the number of patients with the pre‐sarcopenic status increased from five patients (13.1%) before CRT to six (15.8%) after CRT. In addition, one patient (7.7%) was classified with the sarcopenic status only after CRT. According to sex, four male patients with the pre‐sarcopenic status remained pre‐sarcopenic after CRT. In contrast, the number of patients with the pre‐sarcopenic and sarcopenic status increased for female patients only (Table 5).

**TABLE 5:** Changes in the sarcopenic status from pre‐ to post‐neoadjuvant chemoradiotherapy.

Variables	All (*n* = 38)		*p*	Men (*n* = 25)		*p*	Women (*n* = 13)		*p*
	Pre‐CRT	Post‐CRT		Pre‐CRT	Post‐CRT		Pre‐CRT	Post‐CRT	
Sarcopenic status									
Pre‐sarcopenic	5 (13.1)	6 (15.8)	1.00	4 (16)	4 (16)	1.00	1 (7.7)	2 (15.4)	0.59
Sarcopenic	0	1 (2.6)	1.00	0	0	N/A	0	1 (7.7)	1.00
Severely sarcopenic	0	0	N/A	0	0	N/A	0	0	N/A

*Note:* Values in parentheses are percentages, unless indicated otherwise.

Abbreviation: N/A, not applicable.

## 4 Discussion

The present study revealed serial changes in body components, including skeletal muscle mass and associated muscle performance, during preoperative CRT for locally advanced rectal cancer. Despite reductions in body weight and body fat mass, skeletal muscle mass and the majority of skeletal muscle functions were retained during CRT. However, these changes significantly differed between the sexes; body fat mass and non‐predominant HGS decreased only in women, increasing the number of patients with the pre‐sarcopenic or sarcopenic status. These results suggest the need for tailored interventions to build muscle mass and strength during neoadjuvant CRT according to sex.

Limited information is currently available on changes in body composition during CRT. Decreases in HGS, body weight, and body fat mass have been reported during CRT in patients with esophageal cancer, while gait speed remained unchanged [10], which is consistent with the present results.

HGS is a simple and non‐invasive method to assess muscle strength in clinical settings. In patients with cancer, low HGS is an important indicator of malnutrition, impaired cognitive function, and a poor prognosis after surgery [17]. Previous studies demonstrated that a decline in muscular function preceded reductions in muscular mass. HGS is not only a sensitive indicator of short‐term changes in the function of the whole muscle but also reflects the storage of muscle proteins to some extent [18]. Consequently, under invasive treatments such as CRT, changes may occur in HGS before the SMI. Although the mean HGS decline of 1.8 kg in the present study was below the established threshold of > 2.8 kg for a diminished tumor response [19], this reduction represents a clinically relevant change toward early functional deterioration. A significant decrease specifically in non‐dominant HGS was observed in this study. The present results indicate that the prioritized use of the dominant upper limb in daily activities contributed to the preservation of its strength. In contrast, the reduced frequency of use of the non‐dominant limb may have led to the observed decline in grip strength in that specific extremity. In support of this result, a previous study reported a significant reduction of 20% specifically in non‐dominant HGS during chemotherapy for breast cancer [20]. Furthermore, other studies demonstrated that cancer cachexia medications significantly improved non‐dominant HGS [21]. These findings suggest that the non‐dominant side serves as a more sensitive indicator of a patient's overall systemic and functional status.

The phase angle significantly decreased after CRT in both men and women. The phase angle has been proposed as a marker of cellular health, particularly of cell membrane integrity [22]. To date, the phase angle has been suggested as a prognostic, health, nutritional, and functional predictor in clinical settings [23, 24]. It has also been reported to decrease in patients with cancer after chemotherapy [25] and radiation therapy [26], which supports the present results. In addition, low HGS was associated with a decreased phase angle in patients with non‐small cell lung cancer [27], which is consistent with the results obtained herein. The identification of these subtle shifts, particularly a reduction in HGS or a decrease in the phase angle, is crucial for clinicians because they may serve as a sensitive marker for initiating early nutritional support before irreversible sarcopenia occurs.

The ECW/TBW ratio in both sexes slightly increased during the CRT period and then gradually decreased to baseline values by Week 10 (Figure 1E), which may be attributed to a fluid imbalance due to both malnutrition and systemic inflammation [28]. Fluid homeostasis is critically dependent on the dynamics of volume and pressure between the intracellular and extracellular compartments. Hypoalbuminemia compromises colloidal osmotic pressure, which is essential for restricting fluid leakage from the vasculature, consequently leading to an elevated ECW/TBW ratio. Moreover, systemic inflammation induced by cancer‐derived cytokines or cancer treatment may lead to hypervolemic states. However, the clinical implications of the ECW/TBW ratio during CRT remain unknown, and thus need further investigation.

Body weight loss during CRT is common in patients with rectal cancer and has been associated with a low rate of treatment completion and high rate of postoperative anastomotic leakage [29, 30, 31]. Tomoki et al. detected malnutrition, defined as weight loss ≥ 5% after CRT, in 51% of the cohort, and showed that weight loss was associated with a low rate of completion of concurrent chemotherapy with radiation therapy [29]. In the present study, weight loss ≥ 5% at Week 10 (post‐CRT) was observed in 9 patients (23.7%), 8 of whom were women. Furthermore, while the prognostic impact of a longitudinal body fat mass reduction is less clear, a low baseline fat mass is an established indicator of poor outcomes [32]. Fat mass loss during CRT was observed in female patients; however, while the absolute magnitude of this loss may appear modest (a mean decline of 2.3 kg), its clinical significance as a predictor of functional recovery has yet to be fully elucidated. These early changes in body composition may act as a prompt for clinicians to re‐evaluate the patient's nutritional status. Therefore, further investigations are warranted to identify sensitive cut‐off values that will guide early interventions during CRT.

Adverse gastrointestinal events during CRT such as nausea, loss of appetite, diarrhea, and decreased food intake may induce body weight loss. Consequently, a significant decrease in the MNA scores was expected following CRT, along with changes in body composition factors. Contrary to the above, our results showed that BMI, body fat mass, and phase angle decreased significantly, particularly in women, whereas the MNA score decreased only slightly in both men and women after CRT. The MNA is a widely used and recommended tool by the European Society for Clinical Nutrition and Metabolism (ESPEN) for assessing malnutrition in the elderly. This tool incorporates a dietary questionnaire, along with measurements from physical and psychological questionnaires, to assess the nutritional status of geriatric patients. Due to its comprehensive and subjective nature, the MNA may be less sensitive to short‐term, rapid changes, such as those during CRT, highlighting the importance of an objective body composition assessment.

Our results from evaluation with BIA showed that SMI and skeletal muscle mass exhibited a V‐shaped recovery during CRT in the majority of the cohort. Two female patients were classified as having a low muscle mass post‐CRT, suggesting that sex‐specific physiological differences affect the impact of treatment‐induced attrition. While postmenopausal women undergo a significant decline in estrogen, men typically maintain higher testosterone levels, which exert stronger anabolic effects. This sustained androgenic activity in males may facilitate superior body composition restructuring and lead to distinct recovery patterns from females.

Regarding the methodology, the inherent limitations of BIA relative to CT‐based assessments warrant careful consideration. BIA is known to potentially overestimate muscle mass in oncological patients, which may lead to the underdiagnosis of malnutrition [33]. Despite these discrepancies, maintaining longitudinal consistency is paramount for reliable data interpretation. Furthermore, BIA offers unique clinical insights through specialized indices, such as the phase angle or ECW/TBW ratio, which sensitively reflect physiological stress during invasive treatments such as CRT.

Supportive care is essential for both male and female patients because it may significantly affect treatment continuity, therapeutic efficacy, and perioperative outcomes. Female patients generally experience a decline in muscle protein synthesis efficiency (anabolic resistance) due to postmenopausal hormonal changes. In older women and female cancer patients, leucine directly stimulates the mTOR pathway and suppresses muscle atrophy [34]; therefore, the intake of leucine‐rich protein may be highly effective. Additionally, since vitamin D deficiency is closely linked to muscle weakness in women and its supplementation has been shown to improve muscle function and reduce fall risks [35], proactive supplementation needs to be considered. Regarding physical activity, supportive care needs to incorporate not only simple aerobic exercises, such as walking, but also resistance training, even at low intensities. Future interventional studies are warranted to validate these approaches.

This prospective study has several limitations. It was a single‐center study with a small cohort. Furthermore, missing data occurred due to physical adverse events, such as fatigue and anorexia, which were more prevalent in women. However, our sensitivity analysis revealed that the missing data rate was lower in women (23.1%; 3/13) than in men (32.0%; 8/25). Moreover, baseline characteristics were similar between patients with and without missing measurements (Table S1). Although our analysis found no immediate evidence of systematic bias, we acknowledge that attrition bias remains a potential confounding factor due to the complete‐case nature of the longitudinal analysis. Furthermore, as this is a small exploratory cohort with a particularly limited female subgroup, our sex‐based observations are hypothesis‐generating. In addition, due to the limited statistical power of the multivariate analysis, a definitive conclusion regarding the independent effects of sex on body composition changes has yet to be reached. Therefore, any implications regarding the efficacy of sex‐sensitive interventions remain speculative at this stage. Another limitation is that data were not collected on dietary intake and physical activity levels during CRT. This information may help clarify the direct biological effects of CRT and its indirect effects on body composition through lifestyle changes. The diversity of treatment pathways after CRT is another limitation. Specifically, this study included some patients treated with total neoadjuvant therapy and some who received non‐surgical treatment, which prevented a comprehensive evaluation of standardized clinical outcomes such as surgical complications, pathological responses, and long‐term survival.

In conclusion, the present results demonstrate that preoperative CRT may negatively impact body composition and muscle functions, particularly in women. While these sex‐related differences are noteworthy, they should be interpreted with caution due to the limited sample size of the female subgroup. These preliminary findings provide a basis for future research to explore whether sex‐sensitive interventions could potentially influence clinical outcomes. Further large‐scale studies are required to confirm these observations before any definitive clinical recommendations can be made.

## Author Contributions

**Shinya Abe:** conceptualization, methodology, data curation, formal analysis, visualization, writing – original draft, writing – review and editing. **Hiroaki Nozawa:** conceptualization, data curation, writing – review and editing, formal analysis. **Yusuke Taguchi:** data curation, writing – review and editing. **Soichiro Ishihara:** conceptualization, supervision, writing – review and editing, formal analysis, data curation. **Shunsuke Hori:** data curation, writing – review and editing. **Mitsutaka Yakabe:** data curation, writing – review and editing. **Sumito Ogawa:** supervision, writing – review and editing. **Kentaro Abe:** data curation, writing – review and editing. **Kazuhito Sasaki:** data curation, writing – review and editing. **Yumi Yokota:** data curation, writing – review and editing.

## Funding

The authors have nothing to report.

## Disclosure

The authors have nothing to report.

## Ethics Statement

This prospective observational study was approved by the Ethics Committee of the University of Tokyo (No: 2021363NI).

## Consent

Written informed consent was obtained from all participants included in the study.

## Conflicts of Interest

The authors declare no conflicts of interest.

## Supporting information


**Table S1:** Supporting Information.

## Data Availability

The data that support the findings of this study are available from the corresponding author upon reasonable request.
