# Supplementary material for: Sex‐Based Body Composition Changes in Patients With Rectal Cancer Undergoing Preoperative Chemoradiotherapy: A Prospective Observational Study
Source: Ann Gastroenterol Surg. 2026 May 5;10(5):1553–62. doi: 10.1002/ags3.70231 (PMC13394024; doi:10.1002/ags3.70231)
Supplement: Supplementary file 1 [file AGS3-10-1553-s001.docx]

| **Supplemental Table 1 – Comparison between patients with and without missing measurements** | | | | | | |
| --- | --- | --- | --- | --- | --- | --- |
|  |  |  |  | With missing measurements | Without missing measurements |  |
| Variables | |  |  | (n = 11) | (n = 27) | *P* value |
| Patient factors | | |  |  |  |  |
|  | Sex (men) | |  | 8 (72.7) | 17 (63.0) | 0.56 |
|  | Age (years)* | |  | 63 (57 - 72) | 58 (51 - 64) | 0.08 |
|  | BMI (kg/m^2^)* |  |  | 22.4 (18.3 - 23.6) | 23.2 (20.6 - 24.8) | 0.39 |
|  | Charlson comorbidity index | |  |  |  |  |
|  |  | 0 |  | 7 (63.6) | 23 (85.2) | 0.19 |
|  |  | 1 |  | 2 (18.2) | 1 (3.7) |  |
|  |  | 2 |  | 1 (9.1) | 3 (11.1) |  |
|  |  | 4 |  | 1 (9.1) | 0 |  |
| Tumor factors | | |  |  |  |  |
|  | Distance from AV (cm)* | |  | 5.0 (4.0 - 7.0) | 4.0 (3.0 - 6.0) | 0.41 |
|  | Clinical T-stage | |  |  |  |  |
|  |  | cT2 |  | 0 | 1 (3.7) | 0.69 |
|  |  | cT3 |  | 9 (81.8) | 22 (81.5) |  |
|  |  | cT4 |  | 2 (18.2) | 4 (14.8) |  |
|  | Clinical MesoLN | |  |  |  |  |
|  |  | Negative |  | 2 (18.2) | 6 (22.2) | 0.78 |
|  |  | Positive |  | 9 (81.8) | 21 (77.8) |  |
|  | Clinical LLN | |  |  |  |  |
|  |  | Negative |  | 9 (81.8) | 14 (51.9) | 0.08 |
|  |  | Positive |  | 2 (18.2) | 13 (48.1) |  |
|  | Histopathological type | |  |  |  |  |
|  |  | Differentiated (Well/Moderate) |  | 9 (81.8) | 26 (96.3) | 0.16 |
|  |  | Others |  | 2 (18.2) | 1 (3.7) |  |
| Values in parentheses are percentages, unless indicated otherwise | | | | | | |
| BMI; Body mass index, AV; Anal verge | | | | | | |
| MesoLN; Mesorectal lymph node, LLN; Lateral lymph node, | | | | | | |
| *Values are medians (interquartile ranges) | | | | | | |
